# Special Issue: Microbial Nanotechnology

**DOI:** 10.3390/microorganisms12020352

**Published:** 2024-02-08

**Authors:** Kamel A. Abd-Elsalam

**Affiliations:** Plant Pathology Research Institute, Agricultural Research Centre, Giza 12619, Egypt; kamelabdelsalam@gmail.com

## 1. Introduction

Microbial nanotechnology (MN), or microbial nanobiotechnology, is a rapidly expanding research area with the potential to transform various fields, including bioremediation, energy production, medicine, and agriculture [1]. This state-of-the-art technology involves merging nanotechnology with microorganisms to produce innovative materials with distinct properties and functions. For instance, microbial nanotechnology has been utilized to create eco-friendly methods for bioremediating industrial effluents, which can aid in reducing pollution and safeguarding the environment [2]. Moreover, microbial biosynthetic nanoparticles, consisting of a biomolecule capping layer adsorbed on the surface, have been fabricated using microbial nanotechnology. These nanoparticles have demonstrated encouraging results in drug delivery, imaging, and cancer therapy [3]. The immense potential of microbial nanotechnology makes it a stimulating research area with substantial implications for numerous fields.

Microorganisms have evolved remarkable biochemical pathways and mechanisms that enable them to interact with and transform materials at the nanoscale. They possess inherent biological machinery that can produce nanoparticles with precise sizes, shapes, and surface functionalities. These microbial nanoparticles exhibit distinct physical, chemical, and biological properties, making them highly versatile and applicable in various technological domains [4].

One of the primary areas where microbial nanotechnology has shown immense potential is in the synthesis of nanoparticles. Microorganisms can produce nanoparticles through both intracellular and extracellular processes. Intracellular synthesis involves the accumulation of nanoparticles within the microbial cells, whereas extracellular synthesis occurs when microorganisms secrete biocompatible nanoparticles into their environment. These microbial nanoparticles can be synthesized using a wide range of materials, including metals, metal oxides, semiconductors, and organic compounds. The ability to engineer and control the properties of these nanoparticles opens avenues for diverse applications in fields such as medicine, environmental remediation, energy, and electronics [5].

Furthermore, microorganisms can act as living factories to produce nanomaterials with specific functionalities. Through genetic engineering and manipulation, researchers can engineer microorganisms to express proteins or peptides that self-assemble into nanoscale structures. This approach enables the creation of tailored nanomaterials with enhanced properties, such as improved stability, biocompatibility, and targeted interaction with biological systems [6].

Microbial nanotechnology also offers promising possibilities for the development of innovative drug delivery systems. By leveraging the unique properties of microbial nanoparticles, such as their small size, high surface-to-volume ratio, and surface functionalization potential, scientists can design nanocarriers that efficiently transport therapeutic agents to targeted sites within the body. This targeted drug delivery approach enhances treatment efficacy while minimizing side effects associated with conventional drug formulations [7].

In addition to synthesis and drug delivery, microorganisms can play a crucial role in the development of nanosensors, nanoelectronics, and nanodevices. By interfacing microorganisms with nanomaterials, researchers can exploit their inherent biological sensing capabilities to detect and respond to specific environmental or biological signals. This integration of microbial systems with nanotechnology holds promise for applications in environmental monitoring, disease diagnostics, and bioelectronics [8].

As microbial nanotechnology continues to advance, it presents both exciting opportunities and challenges. Ethical considerations, safety assessments, and regulatory frameworks need to be established to ensure the responsible development and deployment of microbial nanotechnology. Additionally, interdisciplinary collaborations among microbiologists, materials scientists, engineers, and medical professionals are essential to drive innovation and maximize the potential of this field [9].

Nanotechnology plays a significant role in multiple aspects of agriculture and food security, including crop development, plant protection, food processing, and the targeted administration of agrochemicals to create disease resistance and boost plant growth. According to research, nanotechnology shows potential for more accurate input applications in the development of these sectors [10]. In conclusion, microbial nanotechnology represents a cutting-edge and rapidly evolving field with vast potential for transforming various sectors. By leveraging the unique capabilities of microorganisms, researchers can synthesize nanomaterials, engineer functional nanodevices, and develop novel applications in areas such as medicine, energy, electronics, and environmental remediation. As we continue to explore and understand the interactions between microorganisms and nanomaterials, microbial nanotechnology is poised to revolutionize numerous fields and contribute to the advancement of science and technology.

## 2. An Overview of Published Articles

One study evaluated the biosynthesis and characterization of selenium nanoparticles (SeNPs) using two distinct endophytic selenobacteria for potential use as biofortifying agents and in various biotechnological applications (contribution 1).

In another contribution, the effects of food-grade titanium dioxide nanoparticles (TiO_2_ NPs), a popular food ingredient, on epithelial permeability, intestinal alkaline phosphatase activity, and nutrient transport across the epithelium were studied using this model. The use of human cells, a synthetic meal, and a bacterial mock community allowed researchers to better understand the effects of dietary changes on small intestinal function, including the microbiota (contribution 2)

Research on non-*Streptomyces* species (i.e., rare actinomycetes) for AgNP production is relatively unexplored. In light of this, another study investigated the biosynthesis of AgNPs for the first time, using two rare actinomycetes, *G. nicotianae* SNPRA1 and L. aridicollis SNPRA2. Additionally, they evaluated the efficacy of these AgNPs as antifungal and anti-mycotoxin agents against mycotoxigenic fungi at non-toxic doses (contribution 3).

To assess the potential of the freshwater snail *B. alexandrina* as a bioindicator for SeONPs, another study investigated the ecotoxicity of naturally synthesized SeONPs in aquatic systems by different aquatic microorganisms. The study evaluated the impact of SeONPs on survival, alanine aminotransferase, and aspartate aminotransferase concentrations, as well as the digestive and hermaphrodite gland structure of *B. alexandrina*. Moreover, in silico molecular docking studies were conducted to analyze the interactions between nano-selenium oxide and ALT/AST. Nanotoxicity was confirmed using *D. magna*, which is a sensitive organism for detecting aquatic environmental pollution (contribution 4).

Additionally, AgNPs were biosynthesized for the first time from the waste leaf extracts of local doum palms in Tabuk, Saudi Arabia, and these demonstrated excellent antibacterial and anticandidal properties against pathogenic strains of bacteria and various yeasts (contribution 5).

TeNPs, produced as intracellular nanorods by *Gayadomonas* sp. TNPM15 isolated from mangrove sediments, were also characterized for their impact on *F. oxysporum* and *A. alternata.* The biosynthesized TeNPs induced notable ultrastructure alterations in the cell wall, exhibiting antifungal potential through inhibitory effects on spore germination and the disruption of membrane permeability and integrity (contribution 6).

Although the use of various microorganisms for the biosynthesis of AgNPs is well documented, there is limited research available on the biosynthesis of AgNPs using actinomycetes. Given this backdrop, another contribution reported that the majority of actinomycetes that produce AgNPs are found in the Streptomyces genus (contribution 7).

Another article explored the synergistic effects of selenium nanoparticles (SeNPs) produced by *Rhizobium pusense* and ciprofloxacin (Cipro) in combating *Acanthamoeba* sp. This combination reduces the sub-lethal dose of ciprofloxacin required and inhibits cyst formation in Acanthamoeba sp. Additionally, this treatment damages the cellular integrity of the protozoa, leading to the leakage of essential proteins, enzymes, amino acids, and sugars. (contribution 8).

In a different research work, a novel approach was developed to self-assemble hybrids of *Shewanella oneidensis* and CdS nanoparticles, resulting in an improved reductive biodegradation capacity. The hybrids were then encapsulated in hydrogels to create a living material that enhanced their recyclability and long-term stability while confining them and protecting them from environmental stress. The living materials developed were found to efficiently biodegrade various organic dyes, including azo and nitroso dyes, through photocatalysis. This study highlights the potential of self-assembled nano-bacteria hybrids for bioremediation and living material development (contribution 9).

Understanding the response of bacteria to metal nanoparticles is crucial in studying the quorum sensing (QS) system of *Pseudomounas. Aeruginosa* and its regulation. Given this knowledge, the exposure of bacteria to low concentrations of bio-AgNPs was found to enhance the expression of QS regulatory genes, affecting functional genes involved in the formation of biofilms and production of virulence factors. As such, this contribution demonstrated that knowledge of bacterial virulence and pathogenicity mechanisms is essential for developing potential targets for anti-virulence therapy (contribution 10).

Furthermore, alginate-based floating microbeads were developed as a promising carrier to enhance the therapeutic index and bioavailability of clarithromycin. The inclusion of therapeutic oils in the microbeads resulted in synergistic effects with this drug, effectively eradicating infections. The synergistic activity produced was assessed through antibacterial assays, comparing the zone of inhibition of the microbeads with both the pure drug and the oils (contribution 11).

In another study, AgNPs were produced using ethyl acetate extracts of *Urtica diocia* (UD) leaves. A characterization of synthesized NPs revealed that the particles were spherical and small, and, because small NPs have desirable therapeutic activities, the NPs synthesized in this study offer promising prospects for development as pharmaceuticals. This characterization investigation revealed the existence of bioactive chemicals in the leaves and that they were incorporated into the AgNPs. (contribution 12).

Additionally, *Trichoderma harzianum* isolates have demonstrated an ability to synthesize a variety of proteins and enzymes without the need for chemical reducers and stabilizers. Biosynthesized AgNPs have shown potential in protecting cotton plants from fungal invasion caused by damping off. However, more research is required to identify the essential bioreducing and biotemplating molecules present in hyphal extracts in order to have greater control over the size and polydispersity of AgNPs (contribution 13).

Another contribution explored π-conjugated polymer nanoparticles (CPNs), given that these nanoparticles are frequently employed as probe materials for bioimaging and medication administration. Moreover, CPN use is on the rise, and they are continually being employed in an increasing number of cutting-edge biomedical sectors due to their specific photophysical and physicochemical characteristics, high compatibility, and ease of functionalization. (contribution 14).

Finally, antimicrobial peptides (AMPs) were investigated as potential alternatives to antibiotics, considering the rising demand for new antimicrobial agents. Derived from microorganisms and widely distributed in nature, AMPs exhibit a broad range of antimicrobial protection, making them suitable for the treatment of infections caused by various pathogenic microorganisms (contribution 15).

## 3. Conclusions

Microbial nanotechnology has several potential applications in the agroecosystem, including the following:

Soil remediation: The utilization of microbial nanotechnology presents a promising approach for the remediation of contaminated soils through the synthesis of nanoparticles that possess the capability to bind to and eliminate pollutants. For instance, microorganisms can synthesize nanoparticles of metals, such as iron, which can be employed to eliminate heavy metals and other contaminants from the soil.

Crop enhancement: Microbial nanotechnology can be used to enhance crop growth and productivity. For example, nanoparticles can be synthesized by microorganisms that can be used as fertilizers, pesticides, or herbicides. These nanoparticles can be engineered to slowly release nutrients or other substances, providing sustained benefits to crops.

Disease control: Microbial nanotechnology can be used to control human and plant diseases by synthesizing nanoparticles that can target and destroy pathogens. For example, nanoparticles of silver, copper, or zinc can be synthesized with microorganisms and used as a natural fungicide or bactericide.

Water management: The utilization of microbial nanotechnology in agriculture has the potential to enhance water management. One way this can be achieved is through the creation of nanoparticles via microorganisms, which have the ability to absorb and gradually release water. As a result, the need for irrigation can be reduced, leading to increased efficiency in water usage and greater sustainability in agricultural practices. Nevertheless, further investigation is necessary to fully comprehend the advantages and potential hazards associated with this technology when applied in agriculture.

Sustainable agriculture: Microbial nanotechnology can help create ecofriendly and sustainable farming methods. Microbial nanotechnology can contribute to the development of a more sustainable agroecosystem by encouraging environmental stewardship, maximizing resource usage, and lowering the use of chemicals.

This Special Issue delves into the use of microorganisms, including bacteria, fungi, and algae, in the production of nanomaterials. However, the wide range of microbiological systems available for use requires standardization in order to produce nanomaterials with consistent and repeatable properties. Given this background, this Special Issue covers the current state of knowledge, mechanisms of microbial synthesis, and challenges in the process. Advancements in biosynthetic pathways and genetic engineering have led to breakthroughs in microbial-based nanosynthesis, which may have potential commercial applications in the creation of sensoristic devices and diagnostic tools, as well as in the control of microbial diseases. Additionally, microbial-generated nanoparticles have shown promise in removing hazardous metals from the environment. To further explore this phenomenon, this Special Issue includes articles examining the sources of nanoparticle exposure and ecotoxicity. Overall, this Special Issue focuses on various aspects of nanomaterials, including microbial synthesis methods, characterization, applications, regulations, nanotoxicity, and challenges, and its principal aim is to present current research on the development of microbial-based nanosynthesis and its potential applications in the biomedical, environmental, and agri-food sectors.
